# Pmp Repertoires Influence the Different Infectious Potential of Avian and Mammalian *Chlamydia psittaci* Strains

**DOI:** 10.3389/fmicb.2021.656209

**Published:** 2021-03-29

**Authors:** Alison Favaroni, Alexander Trinks, Michael Weber, Johannes H. Hegemann, Christiane Schnee

**Affiliations:** ^1^Institute of Molecular Pathogenesis, Friedrich-Loeffler-Institut, Jena, Germany; ^2^Institute of Functional Microbial Genomics, Heinrich Heine University Duesseldorf, Duesseldorf, Germany

**Keywords:** *Chlamydia psittaci*, polymorphic membrane proteins, virulence, egg model, adhesion, mammalian strain, avian strain, infectious potential

## Abstract

*Chlamydia psittaci* is the etiological agent of chlamydiosis in birds and can be transmitted to humans, causing severe systemic disease. *C. psittaci* infects a broad range of hosts; strains are isolated not only from birds but also from mammals, where they seem to have a reduced infectious and zoonotic potential. Comparative analysis of chlamydial genomes revealed the coding sequences of polymorphic membrane proteins (Pmps) to be highly variable regions. Pmps are characterized as adhesins in *C. trachomatis* and *C. pneumoniae* and are immunoreactive proteins in several *Chlamydia* species. Thus, Pmps are considered to be associated with tissue tropism and pathogenicity. *C. psittaci* harbors 21 Pmps. We hypothesize that the different infectious potential and host tropism of avian and mammalian *C. psittaci* strains is dependent on differences in their Pmp repertoires. In this study, we experimentally confirmed the different virulence of avian and mammalian strains, by testing the survival rate of infected embryonated eggs and chlamydiae dissemination in the embryos. Further, we investigated the possible involvement of Pmps in host tropism. Analysis of *pmp* sequences from 10 *C. psittaci* strains confirmed a high degree of variation, but no correlation with host tropism was identified. However, comparison of Pmp expression profiles from different strains showed that Pmps of the G group are the most variably expressed, also among avian and mammalian strains. To investigate their functions, selected Pmps were recombinantly produced from one avian and one mammalian representative strain and their adhesion abilities and relevance for the infection of *C. psittaci* strains in avian and mammalian cells were tested. For the first time, we identified Pmp22D, Pmp8G, and OmcB as relevant adhesins, essential during infection of *C. psittaci* strains in general. Moreover, we propose Pmp17G as a possible key player for host adaptation, as it could only bind to and influence the infection in avian cells, but it had no relevant impact towards infection in mammalian cells. These data support the hypothesis that distinct Pmp repertoires in combination with specific host factors may contribute to host tropism of *C. psittaci* strains.

## Introduction

*Chlamydia psittaci* is an obligate intracellular pathogen of the genus *Chlamydia*. This group of gram-negative bacteria is characterized by a biphasic developmental cycle. The infectious elementary body (EB) adheres to the host cell and is internalized into a vacuole compartment called “inclusion”, where it differentiates into the metabolically active reticulate body (RB). After replication, the RBs re-differentiate into infectious EBs, ready to start another round of infection (Gitsels et al., 2020). EB adhesion to the host cell is a complex multifactorial process and an essential step to establish the infection; however, it is still not fully understood (Hegemann and Moelleken, 2012).

*Chlamydia psittaci* has been identified in more than 465 bird species worldwide and is the etiological agent of avian chlamydiosis (Kaleta and Taday, 2003). The infection can be asymptomatic or it manifests with respiratory symptoms, enteritis and conjunctivitis, eventually leading to systemic and fatal disease. Outbreaks in poultry farms over the years resulted in significant economic losses (Van Loock et al., 2005; Vorimore et al., 2015; Borel et al., 2018). Zoonotic transmission of *C. psittaci* from infected birds is well documented. The pathogen can be transmitted through inhalation of infected aerosol or contact with contaminated feces, causing pneumonia and acute, systemic disease in humans (Harkinezhad et al., 2009). Moreover, the host spectrum of *C. psittaci* is broad. The pathogen has also been isolated from several mammalian hosts, such as cattle and pigs. In these mammals, the infection is mostly latent and the development of clinical disease is possible but rare (Reinhold et al., 2011; Reinhold et al., 2012; Knittler and Sachse, 2015). In contrast to *C. psittaci* from infected birds, *C. psittaci* of mammalian origin does not seem to have a strong zoonotic potential, as only few cases of human transmission from infected mammals have been reported and, in all cases, the primary infection was acquired from infected birds (Hogerwerf et al., 2020). Empiric observations imply that *C. psittaci* strains from mammalian origin (mammalian strains) have a lower infectious and zoonotic potential, compared to strains from avian origin (avian strains) (Knittler and Sachse, 2015).

Genetically, *C. psittaci* is very heterogeneous. Fifteen genotypes based on the immunogenic outer membrane protein (MOMP) have been identified (A to F, E/B, M56, WC, 1V, 6N, Mat116, R54, YP84, and CPX0308), which display a degree of association with host specificity and virulence. Among all *ompA* genotypes, type A, endemic among psittacine birds, is considered to harbor the most virulent and aggressive strains (Sachse et al., 2008; Pannekoek et al., 2010). Interestingly, some mammalian strains have also been classified within genotype A, thus showing that avian and mammalian strains are not necessarily genetically different (Holzer et al., 2020; Shima et al., 2020). To date, very little is known regarding the factors responsible for host specificity and niche adaptation of *Chlamydia* species. Genome analyses highlighted three regions of highest variability among species: (i) the highly variable “plasticity zone,” (ii) the type III secretion system, and (iii) loci encoding members of the polymorphic membrane proteins (Pmps) (Voigt et al., 2012).

Members of the Pmps are found in all *Chlamydia* species, where they represent the largest protein family (Rockey et al., 2000). Pmps are characterized by the presence of multiple repeats of the motifs FxxN and GGA(I,L,V), which are important for adhesion and oligomerization (Molleken et al., 2010; Luczak et al., 2016). Adhesion is their most prominent function, as was shown for all *C. trachomatis* Pmps and representative *C. pneumoniae* Pmps (Molleken et al., 2010; Becker and Hegemann, 2014). In addition, Pmps were proposed to play a role in other infection mechanisms, such as modulation of immune response (Chu et al., 2020). Due to these characteristics, Pmps are considered promising candidates for the development of vaccines, as it was shown for *C. trachomatis* and *C. muridarum* Pmps (de la Maza et al., 2017; Pal et al., 2017). Pmps are divided in six subtypes: A, B/C, D, E/F, G/I, and H and are very heterogeneous, both in numbers and in their sequences (Grimwood and Stephens, 1999). The number of *pmp* genes varies among and within *Chlamydia* species. *C. trachomatis* harbors 9 *pmp* members, while 17 to 21 *pmps* are present in *C. pneumoniae*, 18 in *C. abortus* and 21 in *C. psittaci*, due to an expansion of the E/F and G/I subtypes (Holzer et al., 2020). Differential expression patterns result in further variation; not all Pmps are expressed at the same time, and, within a population, some inclusions are switched off for pmp expression (Tan et al., 2010; Saka et al., 2011; Wheelhouse et al., 2012). Genome comparison of *pmps* from different *Chlamydia* species and strains highlighted the high degree of inter- and intra-species variation among *pmp* genes, with *C. psittaci* displaying one of the greatest and most variable *pmp* repertoire among all *Chlamydia* species (Voigt et al., 2012; Holzer et al., 2020).

Little is known about the specific functions of Pmps in *C. psittaci*. It was shown that *pmps* A, B, D and H are expressed, with PmpA and PmpH being present on the surface of the bacterium. These four Pmps represent rather monolithic subtypes that are the most conserved among *Chlamydia* species, thus suggesting their essential role in *C. psittaci* infection (Goellner et al., 2006; Van Lent et al., 2016a; Van Lent et al., 2016b). However, thus far there is no information concerning the differences of Pmp functions among avian and mammalian *C. psittaci* strains.

In this study, we assess the different infectious potential of avian and mammalian *C. psittaci* strains using an *in vivo* model. We then hypothesize that the different Pmp repertoires expressed by avian and mammalian *C. psittaci* strains influence their infectious and zoonotic potential.

## Materials and Methods

### Cell Culture Conditions and *Chlamydia* Strains

Epithelial buffalo green monkey (BGM) cells and murine fibroblast McCoy cells were grown at 37°C with 5% CO_2_ in Eagle’s Minimum Essential Medium (EMEM) supplemented with 2 mM glutamine and 5% heat inactivated fetal bovine serum (Lonza). Chicken fibroblast UMNSAH/DF-1 (DF1) cells were grown at 37°C with 5% CO_2_ in Iscove’s Modified Dulbecco’s Medium (IMDM), supplemented with Ham’s Nutrient Mixture F-12, 2 mM glutamine and 10% heat inactivated fetal bovine serum.

*Chlamydia psittaci* avian strains 6BC (genotype A) and 09DC77 (genotype B) and mammalian strains 02DC15, 08DC60, C6/98, C19/98, 01DC11 (genotype A), 99DC05 (genotype Mat116), C1/97 (genotype C) and 01DC12 (genotype E) (Table 1) were propagated in BGM cells using standard procedures (Goellner et al., 2006). For functional experiments, two mammalian strains (02DC15 and 08DC60) and two avian strains (6BC and 09DC77) were used. 6BC and 02DC15 have a high number of passages in cell culture, while 08DC60 and 09DC77 have been passaged only few times (Table 1). Chlamydial preparations used in the different infection assays were titrated and stored at −80°C in SPGA (75 g/l sucrose, 0.5 g/l KH_2_PO_4_, 1.2 g/l K_2_HPO_4_, 0.92 g/l L-glutamic acid, 1 g/l bovine serum albumin, pH 7.5), prior use.

**TABLE 1:** Summary of *C. psittaci* strains.

*C. psittaci* strain	Genotype	Host origin	Passage no. in cell culture	GenBank accession no.
02DC15	A	Calf	27	CP002806.1
6BC^#^	A	Parakeet	Unknown	CP002549.1
09DC77*	B	Pigeon	5	NZ_KE356008-NZ_KE356063
08DC60	A	Man	4	CP002807.1
C6/98*	A	Rabbit	8	NZ_KE359921-NZ_KE360062
C19/98	A	Sheep	17	CP002804.1
01DC11	A	Swine	8	CP002805.1
99DC05*	Mat116	Horse	23	NZ_KE356169-NZ_KE356191
C1/97*	C	Sheep	9	NZ_KE359713-NZ_KE359920
01DC12	E	Swine	10	HF545614.1

*Genomes not fully assembled, available in contigs.

*^#^Strain originally obtained from Prof. Daisy Vanrompay (Ghent University, Belgium).*

### Survival and Propagation Tests in Embryonated Eggs

Embryonated chicken eggs (Valo BioMedia) were incubated at 18°C for two days and then at 37.8°C, 60% humidity for nine days with turning eight times a day. After incubation, embryos vitality was checked by candling and eggs were injected at the chorioallantoic membrane site (CAM) with *C. psittaci* preparations (5^∗^10^4^ IFUs) or equivalent amount of mock preparation (day 0) (Jacobsen et al., 2010). The holes were sealed with paraffin and the eggs were incubated at 37.8°C, 60% humidity for eight days. Vitality of the embryos was checked daily by candling, considering two parameters: embryo movement and blood vessels fitness. Embryos death on days 1 and 2 were not considered in the experiments, but accounted to technical problems during inoculation. Vital embryos at day 8 post infection were euthanized by freezing, according to German regulation. Pre-experiments based on previous studies (Braukmann et al., 2012) were performed to select the optimal inoculation dose.

Vitality tests of mammalian *C. psittaci* strains were performed in four rounds for a total of 38 eggs (mock), 40 eggs (02DC15) and 44 eggs (08DC60). Vitality tests of avian strains were performed in three rounds for a total of 52 eggs (mock), 44 eggs (6BC), and 42 eggs (09DC77). Due to the different classification of *C. psittaci* strains, infection with mammalian strains and the relative mock was carried out in laboratory of biosafety level (BSL) 2, while infection with avian strains and the relative mock in BSL3 laboratory.

Eggs from days 6-7-8 after inoculation were selected for propagation analyses. Eggshell was opened and sections of CAM and liver were carefully extracted and frozen at −80°C (Braukmann et al., 2012). After thawing, DNA was extracted, using DNA Roche kit, according to manufacturer’s protocol. *C. psittaci*-specific real-time PCR, targeting the CPSIT_RS03505 gene (Genbank accession number NC_015470) was performed (Angen et al., 2021). *C psittaci* amount was normalized to the number of chicken cells, using real-time PCR, targeting avian actin (Fwd: GATGAAGCCCAGAGCAAAAGA; Rev: TCATC CCAGTTGGTGACAATACC; Probe: HEX-ATCCTGACCCTG AAGTACCCCATTGAACA-BHQ1).

### Expression Analysis of *pmp* Genes via RT-qPCR

#### Total RNA Extraction of *C. psittaci* Infected Cells and cDNA Synthesis

Confluent BGM monolayers, grown in 25 cm^2^ tissue culture flasks, were infected with 10 different *C. psittaci* strains (Table 1) at multiplicity of infection (MOI) of 3 in UltraMDCK medium, supplemented with 1% non-essential amino acid solution and 1% MEM eagle vitamin mixture (Lonza). Infected cells were incubated at 37°C with 5% CO_2_. At 12-24-32 and 48 hours post infection (hpi) total RNA was extracted using RNeasy Mini Kit (Qiagen) and DNA was digested using RNase-free DNaseI (Qiagen), according to manufacturer’s protocols. RNA integrity and absence of DNA contamination were checked via agarose gel electrophoresis and UV-Vis spectroscopy. 1 μg of total RNA was reverse transcribed (Superscript III, Invitrogen), according to manufacturer’s protocol. cDNA was stored at −20°C until further use.

#### Expression Analysis of *pmp* Genes

Expression levels of *pmp1B, pmp2A, pmp4E, pmp6H, pmp8G, pmp17G, pmp19G, pmp21G*, and *pmp22D* genes in 10 *C. psittaci* strains (Table 1) were analyzed by RT-qPCR using Bio-Rad CFX96 system. Specific primers, targeting *pmp*-specific regions identified by the Geneiuos program, were used at a concentration of 100 nM each. Primer efficiencies were calculated by standard curves generated with serial dilutions of cDNA from 02DC15 infected culture at 48 hpi (Supplementary Table 1). The expression of *pmp* genes by quantitative PCR on cDNA samples at 12-24-32 and 48 hpi was performed in technical duplicates using SybrGreen QuantiFast mastermix (Qiagen), with three independent biological replicates. *TyrS* and *gidA* reference genes were used for normalization (Van Lent and Vanrompay, 2016) and data were analyzed with Bio-Rad CFX Maestro software.

### Plasmid Construction and Recombinant Protein Production

#### Cloning of *pmps* and Control Genes

Selected gene fragments of *pmp8G, pmp17G*, and *pmp22D* were amplified from genomic DNA of *C. psittaci* mammalian 02DC15 and avian 6BC strains. A fragment of *omcB* control gene was amplified from *C. psittaci* 02DC15 genomic DNA and *gst* control gene was amplified from pGS-21a (Supplementary Table 2). Amplified gene fragments were cloned into the expression vector pET24a (Novagen), fused with a C-terminal His6-tag, by homologous recombination in *S. cerevisiae* strain CEN.PK2 (Moelleken and Hegemann, 2008). *E. coli* strain XL-1 blue (Stratagene) was used for plasmid amplification. Plasmids were sequenced prior to use.

#### Production of Recombinant Proteins

Expression of recombinant proteins was induced in *E. coli* Rosetta strain (Novagen) with 1 mM IPTG for 4 h. Cells expressing recombinant Pmps and the control protein OmcB were harvested by centrifugation and lysed overnight under denaturing conditions, using buffer containing 6M Guanidine/HCl. Cells insoluble debris were removed by centrifugation at 13’000 rpm for 2 h and the recombinant his-tagged proteins were purified using HiTrap chelating HP columns (GE Healthcare) in buffer containing 6M Urea and 500 mM Imidazole. Pmp17G and Pmp22D from *C. psittaci* strains 02DC15 and 6BC, and OmcB were renatured by dialysis at 4°C against PBS at pH 7.4. Pmp8G from *C. psittaci* strains 02DC15 and 6BC were renatured using Amicon Ultra-15 Centrifugal Filter Units columns (Merck), by centrifugation at 3’500 rpm for 3–5 h at 4°C in PBS containing 20–100 mM L-Arginine (Chen et al., 2008). Recombinant His-tagged GST protein was purified under native conditions, as previously described (Fechtner et al., 2013). Renatured and native proteins were stored at −80°C and centrifuged at 10’000 g for 15 minutes at 4°C prior use. Purity and identity of purified recombinant proteins were verified by SDS-PAGE and immunoblot.

### SDS-PAGE and Immunoblotting Analysis

SDS-PAGE and immunoblotting analyses were performed as previously described (Sambrook and Maniatis, 1989). Purified recombinant his-tagged proteins and cellular actin were detected using monoclonal anti-histidine and anti-β actin antibodies, respectively (Sigma) and visualized with AP-conjugated antibodies (Promega). SDS-PAGE gels were stained with Coomassie Brilliant Blue G250 (Serva).

### Adhesion Assay With Soluble Proteins

Confluent BGM, McCoy and UMNSAH/DF-1 monolayers grown in 24-well plates were incubated with 200 μg/ml soluble recombinant proteins for 1 h at 37°C. Unbound protein was removed by washing the cells three times with PBS. Cells and bound proteins were detached with cell dissociation solution (Sigma) and analyzed by immunoblotting. Anti-histidine antibody was used to determine the input of soluble recombinant proteins and their binding capacity, anti-β actin antibody was used to verify the amount of cells in each sample. The intensity of western blot bands was measured with ImageJ and the intensity of adhesion of each protein was expressed as a percentage of the intensity of the respective band in the input loading control blot.

### Infection Blocking Assay With Soluble Proteins

Confluent BGM, McCoy and UMNSAH/DF-1 monolayers grown in tubes containing microscopy slides, were incubated with 200 μg/ml soluble recombinant proteins for 1 h at 37°C. Unbound protein was removed by washing the cells three times with PBS. Cells were then infected with *C. psittaci* 6BC, 09DC77, 02DC15, and 08DC60 preparations at the selected MOI in cell culture medium and incubated at 37°C with 5% CO_2_. After 2 h, medium was replaced with 1 ml of UltraMDCK medium, supplemented with 1% non-essential amino acid solution and 1% MEM eagle vitamin mixture and the infection was carried out for 30–48 h, according to the cell-strain combination. BGM and McCoy cells were infected with *C. psittaci* 02DC15 and 08DC60 (MOI 10) and with *C. psittaci* 6BC and 09DC77 (MOI 5) for 48 h. UMNSAH/DF-1 cells were infected with *C. psittaci* 02DC15 and 08DC60 (MOI 50) for 48 h and with *C. psittaci* 6BC and 09DC77 (MOI 3) for 48 and 30 h, respectively. Infected cells were then fixed with methanol and chlamydial inclusions were detected with FITC-labeled monoclonal antibody (IMAGEN, Thermo). Inclusions were visualized using an Olympus BX41 microscope, pictures of 10 random visual fields were taken for each sample with a Leica DFC450C camera and analyzed using ImageJ. Inclusion numbers were expressed as a percentage of the number of inclusions determined by the PBS-treated samples.

### Bioinformatics and Statistical Analyses

Sequences of all *pmps* genes from 10 different *C. psittaci* strains were identified by alignments with known annotated *pmps* sequences from *C. psittaci* 6BC and 02DC15 fully assembled genomes, using alignment programs Blast and Geneious (Altschul et al., 1990). *C. psittaci* 6BC, 02DC15, 08DC60, C19/98, 01DC11 and 01DC12 genomes are fully assembled, while *C. psittaci* 09DC77, C6/98, 99DC05 and C1/97 genomes are available as contigs (Table 1). In order to identify the most variable *pmps*, all *pmps* from all the analyzed *C. psittaci* strains were aligned against the respective *pmp* from *C. psittaci* 02DC15, used as reference strains.

Expression heatmaps were generated using the R package pheatmap v1.0.8. The statistical tests used for each assay are indicated in the figure legends.

## Results

### Avian *C. psittaci* Strains Are More Virulent Than Mammalian Strains in a Chicken Embryo Model

**FIGURE 1:**
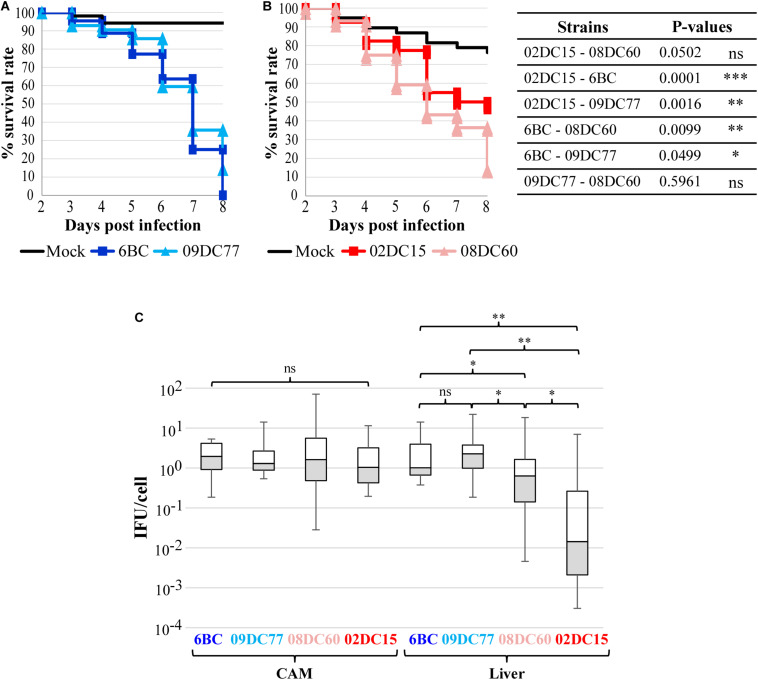
Avian *C*. *psittaci* strains have a higher virulence in chicken embryos than mammalian *C. psittaci* strains. **(A,B)** Kaplan–Meier survival curves of chicken embryos challenged with *C. psittaci* strains. Embryonated eggs were infected with 5*10^4^ IFU/egg on developmental day 10 and their survival was recorded for 8 days. **(A)** Survival curves of eggs infected with avian *C. psittaci* strains 6BC (*n* = 44), 09DC77 (*n* = 42) and mock (*n* = 52). **(B)** Survival curves of eggs infected with mammalian *C. psittaci* strains 02DC15 (*n* = 40), 08DC60 (*n* = 44) and mock (*n* = 38). P-values were calculated using Chi-square tests at day 8, to highlight the different virulence of the strains at the end of the infection. *p < 0.05, **p < 0.01, ***p < 0.001 **(C)** Dissemination of *C. psittaci* strains in the CAM and liver of infected embryonated eggs at the end of the experiment. *C. psittaci* genomic DNA was quantified by specific real-time PCR and normalized to the number of chicken cells. The amount of *C. psittaci* in CAM and liver is displayed in boxplots. P-values were calculated using Mann–Whitney U test. *p < 0.05, **p < 0.01.

Using a chicken embryo infection model, we focused on verifying the assumption that avian and mammalian *C. psittaci* strains differ in their infectious potentials. For this purpose, two avian strains (6BC and 09DC77) and two strains of mammalian origin (02DC15 and 08DC60) (Table 1) were used to inoculate embryonated eggs at the chorioallantoic membrane (CAM). The vast majority of eggs infected with the two avian strains survived until day 6 post infection (p.i.), after which the number of viable eggs started to drop considerably, indicating that at this time point chlamydiae have spread through the CAM and reached the embryos. On day 8 p.i., the last day of the experiment, all 44 embryos infected with 6BC had died (survival rate of 0%) and only 14% of 09DC77 infected eggs were still viable, while the mock-infected eggs had a survival rate of 94% (Figure 1A). Eggs infected with strain 08DC60 isolated from a human case of psittacosis showed a high survival rate until day 5, which dropped to 13% on the last day of the experiment, while the mock-infected eggs in this experimental set-up had a survival of 76%. Considering the respective mock-infected eggs, 09DC77 had a final survival rate of 12%, while 08DC60 of 20%. Interestingly, even though the survival rate of embryos infected with the mammalian strain 02DC15 also started to decrease on day 5 p.i., the survival rate at the end of the experiment was 47% (65%, compared to the mock-infected eggs at day 8), which is considerably higher than those of all other infected groups (Figure 1B).

Dissemination analysis using qPCR amplification of chlamydial DNA showed that chlamydiae spread through the CAM into the embryos in all inoculated eggs. Chlamydia load in the CAM was comparable among all strains, while bacterial dissemination to the liver was significantly higher in eggs infected with the avian strains, in comparisons to eggs infected with the strains from mammalian origin. Interestingly, the bacterial load in the liver was significantly lower in the eggs infected with the mammalian strain 02DC15, in comparison to those infected with the human isolate 08DC60 (Figure 1C).

It is noteworthy that the direct comparison between the genetically almost identical genotype A strains 02DC15 and 6BC confirms the higher dissemination and killing rate of the avian strain.

### Sequence Variability of *C. psittaci pmp* Genes in Reference to Host and Genotype

**FIGURE 2:**
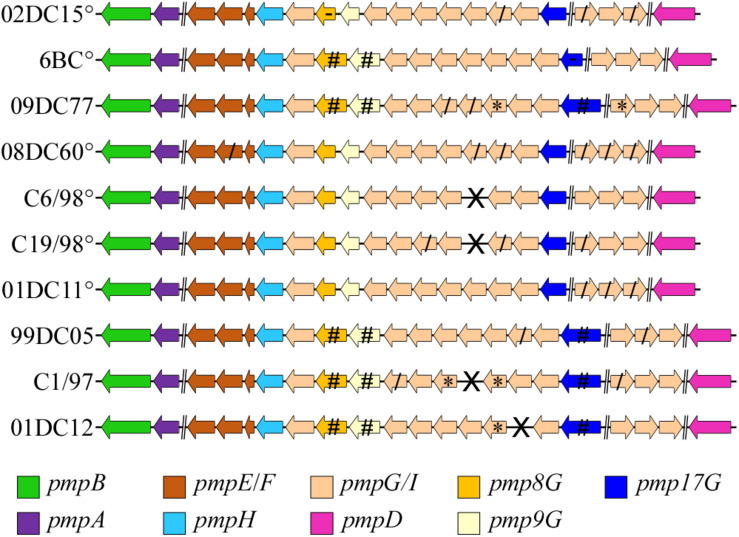
Genomic organization of all identified *pmp* genes of *C. psittaci* strains in the study. °: *C. psittaci* strains belonging to genotype A. Brake (**//**) represents a genomic region bigger than 5000 bp (implemented from Van Lent et al., 2016a). X: missing gene, *: Partial sequence, due to a genome sequence or assembly gap, /: truncated gene product. *Pmp8G, pmp9G*, and *pmp17G* genes (highlighted in dark yellow, light yellow, and blue, respectively) represent the most diverse genes according to their size among the different strains. **#**: genes 350, 680, and 1200 bp bigger than the respective *pmp8G, pmp9G*, and *pmp17G* from 02DC15, used as reference strain. -: The shortest *pmp8G* and *pmp17G* genes among the respective genes in all strains.

As Pmps are considered one of the major source of variability between and within *Chlamydia* species, we selected 10 *C. psittaci* strains (six genotypes A and one of each genotypes B, C, E, and Mat116) (Table 1) and identified all *pmp* genes present. Each *pmp* sequence from each strain was compared to the respective *pmp* of *C. psittaci* mammalian strain 02DC15. The latter, isolated from an aborted calf, was selected as reference strain due to its fully assembled genome, its widespread use in laboratory experiments and, most importantly, its genome being almost identical to the genome of avian strain 6BC (Holzer et al., 2020; Shima et al., 2020). Six out of 10 strains harbored 21 *pmps*, while 20 *pmps* were found in the remaining four strains. *Pmp14G* is missing in C6/98 and C19/98 strains (genotype A); C1/97 (genotype C) lacks *pmp13G*, and *pmp15G* is missing in 01DC12 (genotype E). Since C6/98 and C1/97 genomes are not completely assembled, the lack of *pmps* might also be due to gaps left by the assembly process (Figure 2). In general, the genomic organization of all *pmps* is conserved in all strains analyzed and their sequences share a high degree of similarity. For instance, the conserved *pmp22D* has a range of identity to 02DC15 *pmp22D* of 92-100%, while more heterogeneous *pmp19G* has a range of 82-99% (Supplementary Table 3). *Pmp* genes within genotype A strains are highly conserved, regardless of host origin. As expected, the greatest variation, reflected in gene length, has been found within the *pmpG* group, in particular *pmp8G, pmp9G*, and *pmp17G* genes. All three genes are almost identical in all mammalian strains of genotype A, while their size is much larger in strains outside genotype A, including avian strain 09DC77 (genotype B) and mammalian strains of genotypes C, E and Mat116. Interestingly, *pmp8G* and *pmp9G* from genotype A strain 6BC are similar in size to the respective genes of non-genotype A strains. Moreover, 6BC harbors the shortest version of *pmp17G* and 02DC15 the shortest version of *pmp8G*, among all analyzed strains (Figure 2 and Supplementary Table 3).

### *PmpG*s Are the Most Variably Expressed Pmps Among *C. psittaci* Strains

**FIGURE 3:**
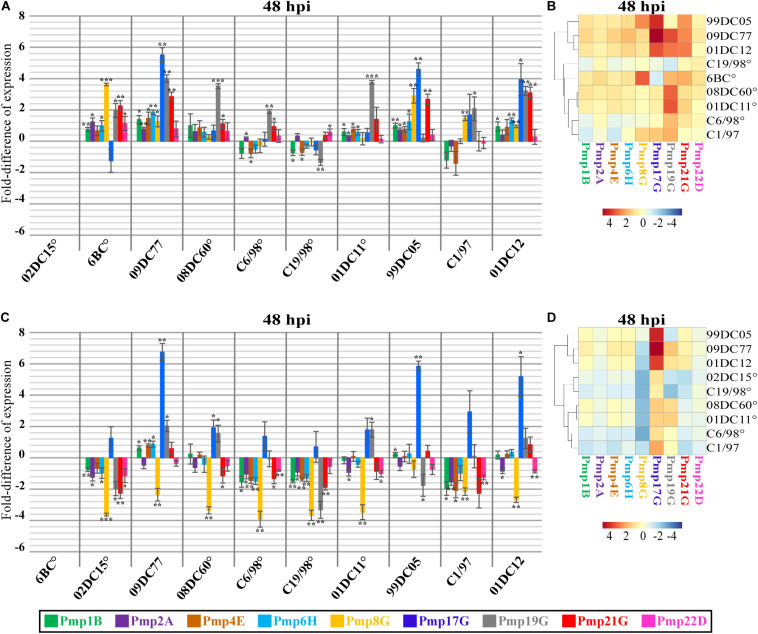
Expression profiles of *pmp* genes in different *C. psittaci* strains infecting mammalian epithelial (BGM) cells at 48 hpi. Transcript levels of different color-coded *pmp* genes were measured by RT-qPCR at 48 hpi and expressed as relative fold-difference, compared to the respective *pmp* gene of mammalian 02DC15 **(A,B)** or avian 6BC **(C,D)** reference *C. psittaci* strains.°: *C. psittaci* strains belonging to genotype A. Relative expression levels represent the mean of three independent biological replicates (*n* = 3). **(A–C)** Fold-difference of relative expression for each *pmp* in each strain is shown in color-coded columns, with the error bars representing the standard deviation of the mean. *p*-values were calculated using One-way ANOVA and *post hoc* t-test. *p < 0.05, **p < 0.001, ***p < 0.0001. **(B–D)** Expression heat maps illustrate the fold-difference of relative expression for each *pmp* in each strain. The strains are grouped according to their *pmp* expression pattern relative to those of 02DC15 or 6BC. Shades of red-colored cells represent folds of expression higher than the respective *pmp* gene in the reference strains, while shades of blue-colored cells represent folds of expression lower than the respective *pmp* gene in the reference strains.

The expression profiles of nine different *pmps*, representative of all subtypes (*pmps 1B, 2A, 4E, 6H, 8G, 17G, 19G, 21G*, and *22D*), were investigated in epithelial BGM cells at the middle (12–24 hpi) and late stages (32–48 hpi) of the infection cycle of avian and non-avian *C. psittaci* strains. Expression levels of each *pmp* gene were compared to the expression of the corresponding *pmp* in reference mammalian 02DC15 and avian 6BC strains (Figure 3 and Supplementary Figures 1–3).

Clustering of non-genotype A avian and mammalian strains could be observed at all time points based on the expression of all analyzed *pmps*. In particular, when compared to the mammalian strain 02DC15, *pmps* of the G group (*8G, 17G, 19G* and *21G*) displayed upregulation in avian 6BC and 09DC77 as well as in non-genotype A mammalian 99DC05, C1/97 and 01DC12 strains. Similarly, when compared to the avian strain 6BC, all strains shared a similar pattern of downregulation of *pmp8G*, and avian 09DC77 and mammalian non-genotype A strains showed upregulation of *pmp17G*, mostly during the late cycle. All mammalian strains belonging to genotype A exhibited similar expression of all analyzed *pmps*, with the only exception of *pmp19G*, which was slightly overexpressed in 01DC11 and 08DC60 (Figure 3 and Supplementary Figures 1–3).

Looking more closely at the various *pmps*, it was observed that *pmps 1B, 2A, 4E, 6H* and *22D* were expressed at similar levels (below 2-fold difference) in all strains during the infection cycle, regardless of host origin or genotype (Figure 3 and Supplementary Figures 1–3). On the other hand, selected members of the *pmpG* group presented different expression patterns. During mid-infection (12–24 hpi), *pmp19G* showed upregulation in avian 09DC77 and mammalian 01DC12 (genotype E) (Supplementary Figures 1,2). Beginning at 24 hpi and getting more prominent in the late infection phase (32–48 hpi), 6BC displayed upregulation of *pmp8G*, compared to all other strains, except for mammalian 99DC05 (genotype Mat116). At late infection, both 6BC and 02DC15 presented lower levels of *pmp17G* expression, compared to avian 09DC77 and non-genotype A mammalian strains, in which the levels of *pmpGs* showed a tendency of slight upregulation (Figure 3 and Supplementary Figure 3).

In general, while a clear host-specific (avian vs. mammalian) expression pattern was not evident in our set-up, our data show that members of the G group are the most variably expressed Pmps among *C. psittaci* strains. Especially *pmp8G* and *pmp17G* present variable expression not only among genotypes, but also between avian and non-avian strains.

### Recombinant Pmp22D, Pmp8G, and Pmp17G Mediate Differential Adhesion to Mammalian and Avian Cells

**FIGURE 4:**
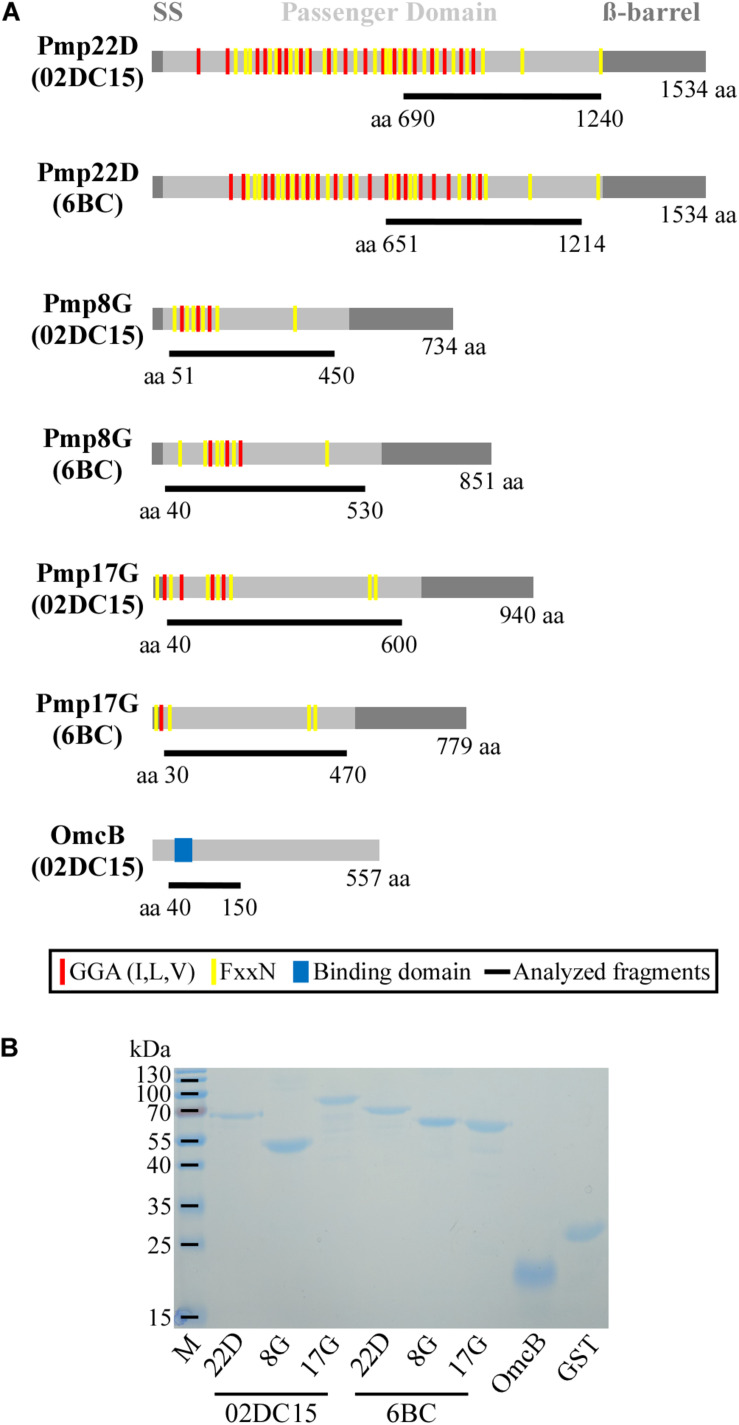
Selected proteins. **(A)** Schematic representation in scale of Pmp22D, Pmp8G, and Pmp17G from *C. psittaci* mammalian strain 02DC15 (GenBank accession numbers AEG87787.1, AEG87282.1, AEG87291.1) and avian strain 6BC (GenBank accession numbers ADZ18878.1, ADZ18372.1, ADZ18350.1). Gray boxes indicate the characteristic autotransporter structure of Pmps, with the N-terminal signal sequence (SS), the central passenger domain (PD) and the C-terminal ß-barrel. Repeated motifs GGA (I,L,V) and FxxN are indicated by red and yellow lines, respectively. OmcB from *C. psittaci* 02DC15 (GenBank accession number AEG87193.1), used as positive control, is indicated with a gray box and the functional binding domain “AKKVRF” shown in blue, according to Fechtner et al. (2013). The protein fragments analyzed in this study are indicated as black lines below the respective proteins with their amino acid positions and carry a C-terminal His6-tag. **(B)** Coomassie stained SDS-PAGE of 1 μg renatured recombinant His-tagged Pmps and control recombinant OmcB and GST proteins.

Due to their genetic and expression characteristics, we investigated Pmp8G and Pmp17G further for their role in host tropism of avian and non-avian *C. psittaci*. For functional tests, Pmp8G and Pmp17G were selected as “variable” Pmps and Pmp22D as “conserved” Pmp. Fragments of their passenger domains were selected from the reference strains avian 6BC and mammalian 02DC15 (Figure 4A and Supplementary Table 2), allowing direct comparison between genetically related avian and mammalian genotype A strains. His-tagged rPmps were produced in *E. coli*, purified via affinity chromatography and analyzed using SDS-PAGE, prior to use. Recombinant his-tagged GST was produced as negative control and recombinant his-tagged OmcB from 02DC15, harboring a domain with a conserved glycosaminoglycan binding sequence (Fechtner et al., 2013), was used as positive control (Figure 4B and Supplementary Table 2).

**FIGURE 5:**
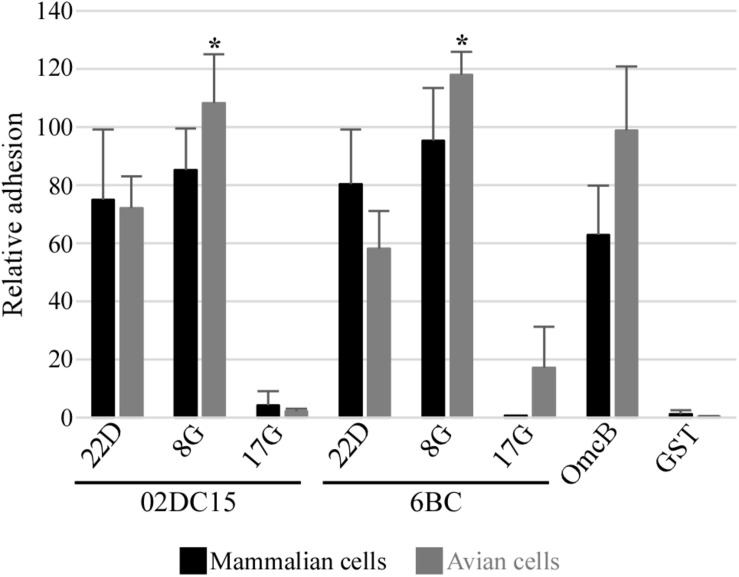
Adhesion ability of different Pmps from mammalian and avian *C. psittaci* strains. Adhesion ability of 200 μg/ml soluble recombinant Pmp22D, Pmp8G, and Pmp17G from mammalian *C. psittaci* 02DC15 and avian 6BC strains. Recombinant OmcB and GST were used as positive and negative control, respectively. Comparison of adhesion intensity of the different rPmps and control proteins to confluent McCoy mammalian fibroblasts (black) and UMNSAH/DF-1 avian fibroblasts (gray). Adhesion abilities were evaluated by immunoblotting and the intensity of the bands was measured with ImageJ and expressed as a percentage of the input loading band of the respective protein. Relative adhesion represents the mean of three independent replicates (*n* = 3). *p*-values were calculated using paired *t*-test, by comparing the relative adhesion of each Pmp to the two cell lines. *p < 0.05.

Adhesion abilities of mammalian and avian rPmp8G, rPmp17G, and rPmp22D to mammalian and avian cells were analyzed. Recombinant proteins were incubated for 1 h with mammalian and avian fibroblasts (McCoy and UMNSAH/DF-1). Adhesion to mammalian epithelial cells (BGM) was also performed as control. Recombinant Pmp22D and Pmp8G from both avian and mammalian *C. psittaci* strains were able to adhere strongly to both mammalian and avian cells, similarly to rOmcB. Interestingly, while rPmp17G from mammalian origin could not bind to either avian or mammalian cells, avian rPmp17G could also not adhere to both mammalian cell lines, but showed a weak adhesion to avian cells (Figure 5 and Supplementary Figure 4), suggesting a role of Pmp17G in differential host tropism of *C. psittaci* strains.

### Recombinant Pmp22D, Pmp8G, and Pmp17G Have Different Relevance for Infection of Avian and Mammalian *C. psittaci* Strains

Finally, the influence of recombinant Pmp22D, Pmp8G, and Pmp17G on infection rates of *C. psittaci* strains in mammalian and avian cells was explored. Recombinant Pmp22D, Pmp8G, and Pmp17G from mammalian 02DC15 and avian 6BC *C. psittaci* strains and control proteins were pre-incubated for 1 h with mammalian epithelial cells (BGM) and with mammalian and avian fibroblasts (McCoy and UMNSAH/DF-1), prior to infection with avian (6BC, 09DC77) or mammalian (02DC15, 08DC60) *C. psittaci* strains. We hypothesized that, if the proteins bound to receptors relevant for infection, this would block one of the infection pathways used by chlamydial particles, resulting in a lower infection rate. Adhesive rOmcB and non-adhesive rGST were used as positive and negative controls, respectively.

**FIGURE 6:**
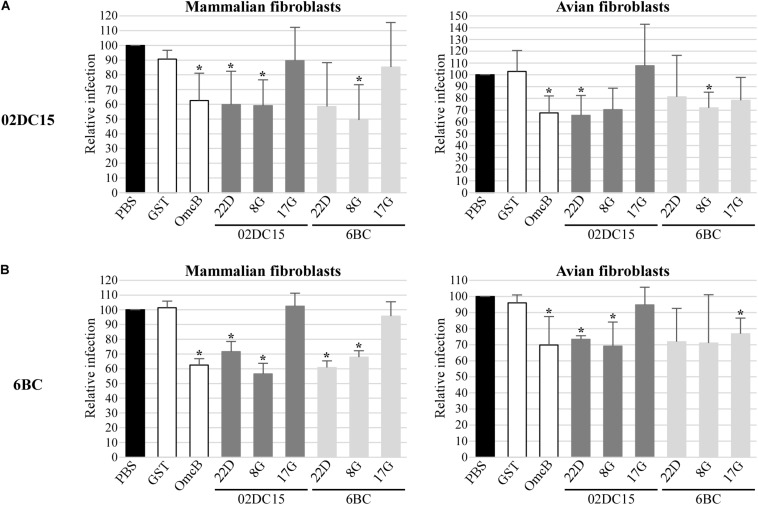
Pmps from mammalian and avian *C. psittaci* strains show different relevance for *C. psittaci* infection. Mammalian (McCoy) and avian (UMNSAH/DF-1) fibroblasts were pre-incubated with PBS (black), controls recombinant GST and OmcB (white), or recombinant Pmps from mammalian 02DC15 (dark gray) and avian 6BC (light gray) *C. psittaci* strains for 1 hour prior to incubation with *C. psittaci* mammalian 02DC15 **(A)** or avian 6BC **(B)** strains. The numbers of inclusions were determined by observation of 10 microscopy fields for each condition. The infection rate for each sample is expressed as a percentage of the relative PBS-treated sample, set to 100%. The relative infection rates represent the mean of three independent experiments (*n* = 3). *p*-values were calculated using paired *t*-test. *p < 0.05.

Our data show that pre-incubation of avian and mammalian cells with rOmcB and with avian and mammalian rPmp22D and rPmp8G could moderately to strongly reduce the infection in a range of approximately 20 to 60%, compared to the mock (PBS) treated cells. Indeed, adhesive rPmp22D, rPmp8G and rOmcB were able to block the infection of all avian and mammalian *C. psittaci* strains analyzed in both avian and mammalian fibroblasts (Figure 6 and Supplementary Figure 5), as well as in mammalian epithelial cells used as control (Supplementary Figure 6). These findings indicate that Pmp22D, Pmp8G and OmcB are adhesins, essential for the infection process of *C. psittaci* strains in general.

On the other hand, pre-incubation of all cells with non-adhesive mammalian rPmp17G resulted in no or only minor reduction of *C. psittaci* infection (Figure 6 and Supplementary Figures 5, 6). Strikingly, pre-incubation of both mammalian cell lines with avian rPmp17G resulted in no or minor reduction of the infection rate of all *C. psittaci* strains analyzed. In contrast, pre-incubation of avian cells with avian rPmp17G could moderately reduce the infection of all avian and mammalian *C. psittaci* strains by approximately 20-30% (Figure 6 and Supplementary Figures 5, 6), suggesting that Pmp17G might bind receptors specific for avian cells.

## Discussion

It is an accepted concept that *C. psittaci* strains from avian and mammalian origin harbor different intrinsic characteristics, with avian strains being considered to have a higher infectious and zoonotic potential (Knittler and Sachse, 2015). While this statement is the result of years of empiric observations, there has been no experimental proof so far.

To address the varying infectious potential among *C. psittaci* strains, we directly compared the infectious ability of four strains of different origin, two avian strains (6BC and 09DC77) and two strains isolated from mammals (02DC15 and 08DC60). The chicken embryo is a valuable and safe *in vivo* model that had been used to investigate virulence of bacterial, fungal and viral pathogens, including *Chlamydia* species (Braukmann et al., 2012; Andersson et al., 2015; Filcek et al., 2019). Inoculation of chlamydiae at the chorioallantoic membrane (CAM) resembles a natural infection route across epithelial cells and is able to elicit a pronounced inflammatory response (Braukmann et al., 2012). Using this system, avian *C. psittaci* strains indeed proved to be more virulent than mammalian strains. Avian strains 6BC and 09DC77 had the highest dissemination rate within the embryos and caused higher embryo mortality than mammalian strain 02DC15. To give more strength to these data, it is important to remember that mammalian 02DC15 is genetically very close to avian 6BC (Holzer et al., 2020; Shima et al., 2020), still they have a different infectious potential in our embryonated egg model. This direct comparison between genetically related 6BC and 02DC15 strains in an *in vivo* model serves as proof of principle for the different infectious potential between avian and mammalian strains.

In contrast, the mortality caused by the human isolate 08DC60 was similar to that caused by avian strain 09DC77, and its dissemination rate was higher than that of mammalian 02DC15. 08DC60 is a human isolate of a psittacosis case, meaning that the original infection source was a bird. Indeed, many psittacosis cases trace back to 6BC and genetically equivalent strains. There is no evidence for transmission to humans of genetically related *C. psittaci* mammalian strains, such as 02DC15. Strain 02DC15 is able to infect non-human mammals, such as cattle, causing no or mild symptoms and it can be transmitted to naive calves in experimental settings (Ostermann et al., 2013). Zoonotic transmission of *C. psittaci* from infected mammals apparently is a rare event; only a handful of cases are reported. For instance, five psittacosis cases were identified in individuals exposed to abortion material of a mare in Australia (Branley et al., 2016). The mare was obviously passing the highly virulent *C. psittaci* strain 6BC, identical to the 6BC strain present in Australian parrots (Chan et al., 2017; Jelocnik et al., 2017). In general, mammals seem to be an accidental host of avian *C. psittaci* strains (Harkinezhad et al., 2009; Hogerwerf et al., 2020). One could argue that all strains isolated from mammals were originally acquired from birds and were therefore “avian strains,” which evolved to “mammalian strains”. Clearly, a single or few passages in the mammalian host are not enough to attenuate an avian strain, as for example the one transmitted to humans from the mare in Australia, but a more intimate interaction and possibly more passages in the host may be required. Consequently, we propose a distinction between *C. psittaci* “mammalian isolates of avian strains” (such as 08DC60) and fully adapted “mammalian strains” (such as 02DC15).

It is still unclear what makes the difference between a mammalian host shedding the pathogen and controlling the infection by attenuating the avian strain’s virulence and a dead-end mammalian host as encountered in most human psittacosis cases. The host-pathogen interaction may have an influence not only on the outcome of infection (Knittler et al., 2014) but also on the intrinsic characteristics of the pathogen.

Host adaptation is achieved by pathogens using many different molecular mechanisms, such as the ability to adhere to certain cells, the production of toxins, the ability to interfere with the immune response and many more (Baumler and Fang, 2013). Many studies focused on identifying genetic markers or virulence factors involved in host tropism among *Chlamydia* species. Recently, Holzer et al. compared 33 strains from 12 *Chlamydia* species, with the aim of identifying genomic traits specific for adaptation to avian hosts, including both avian and non-avian *C. psittaci* strains. According to this study, *C. psittaci* genomes are very heterogeneous; but no unambiguous genetic marker or virulence factor was identified that could be used to distinguish between avian and non-avian *C. psittaci* strains (Holzer et al., 2020).

Polymorphic membrane proteins (Pmps) are highlighted as one of the most relevant sources of heterogeneity among and within species (Holzer et al., 2020). Pmps are a family of proteins found only in *Chlamydia* species and are predicted autotransporters, with the β-barrel able to enter the outer membrane while releasing the functional passenger domain into the extracellular space (Henderson and Lam, 2001). *C. psittaci* harbors 21 Pmps, due to the expansion of the E/F and G/I subtypes, and genomic studies showed that they represent the most variable *pmps* among *Chlamydia* species (Voigt et al., 2012; Van Lent et al., 2016a; Holzer et al., 2020). In our study, we could identify all *pmp* genes from 10 avian and non-avian *C. psittaci* strains belonging to different genotypes and the highest variation was found among members of the G subtype, in particular among *pmp8G, pmp9G*, and *pmp17G*. In general, even though *pmp* sequence variability seems to be mostly dependent on genotype, genotype A avian strain 6BC shares some characteristics with non-genotype A strains. In particular, *pmp8G, pmp9G*, and *pmp17*G genes seem to be conserved in some strains, rather than in others.

Polymorphic membrane proteins variability is not only a consequence of heterogeneous sequences and copy numbers, but also of differential expression patterns. For instance, each Pmp seems to have its own pattern in the course of the developmental cycle, and some Pmps even show inconsistent expression within the same population of chlamydiae (Tan et al., 2010; Saka et al., 2011; Wheelhouse et al., 2012). While regulation of Pmp expression is still unexplored, this variable expression resembles phase variation, a known mechanisms used by several pathogens to variably express certain antigens in order to evade host recognition and immune response (van der Woude and Baumler, 2004). Different expression patterns of individual Pmps were also shown for another avian *C. psittaci* strain (Van Lent et al., 2016a). Our expression analysis of nine representative *pmps* (*pmp 1B, 2A, 4E, 6H, 8G, 17G, 19G, 21G*, and *22D*) in 10 *C. psittaci* strains during middle and late cycle revealed that members of the PmpG group proved to be the most variably expressed Pmps among all strains. While we could not identify a clear avian and mammalian expression profile, we were able to show that *pmp8G* and *pmp17G* were expressed differently among genotypes and among avian and non-avian strains. A limitation of our set-up is that all the expression studies were performed on a mammalian epithelial cell line (BGM). It remains unclear whether expression studies performed on avian cells would influence *pmp* expression profiles of individual strains. However, our study serves as a proof of principle and as an indication that certain *pmp* genes, especially members of the G group, show differences in expression that are probably related to the host and/or genotype.

Polymorphic membrane proteins are generally considered adhesins, involved in the intimate attachment of the bacterial particles to the host cells. Pmps from *C. trachomatis* and *C. pneumoniae* are confirmed adhesins, instrumental for infection of human cells (Molleken et al., 2010; Becker and Hegemann, 2014). Interestingly, it was demonstrated that *C. pneumoniae* Pmp21D is able to bind the epidermal growth factor receptor (EGFR), but other *C. pneumoniae* Pmps and the PmpD homolog in *C. trachomatis* could not, suggesting that different Pmps might interact with different receptor structures on the host cells, probably having a redundant function (Molleken et al., 2013). Much less is known about *C. psittaci* Pmps: it was shown that Pmps A, B, D and H are expressed, and PmpA and PmpH are found at the bacterial surface, but no functions have been assigned to the proteins yet (Goellner et al., 2006; Van Lent et al., 2016a). In this study, we focused on investigating the function of selected Pmps in host tropism of *C. psittaci* strains. First, we selected two variably expressed Pmps (Pmp8G and Pmp17G) and one stably expressed Pmp (Pmp22D) and produced them from the genetically related reference avian 6BC and mammalian 02DC15 strains. “Avian” and “mammalian” Pmp22D are 100% identical, while Pmp8G and Pmp17G have different sizes, with Pmp8G being larger in avian strain 6BC and Pmp17G being larger in mammalian strain 02DC15. Even though Pmp8G and Pmp17G have different sizes in the two strains, the overlapping sequence is 100% identical. In addition, we selected OmcB from *C. psittaci* strain 02DC15 as positive control. OmcB is a conserved protein, shown to be involved in the adhesion process of *C. pneuominae*, *C. trachomatis* LGV serovars and *C. caviae* (Ting et al., 1995; Fechtner et al., 2013). However, the role of OmcB in the adhesion process of *C. psittaci* has not been proven so far. OmcB from 02DC15 harbors a domain with a conserved glycosaminoglycan binding sequence, identified as responsible binding domain in *C. pneumoniae* (Fechtner et al., 2013).

In our setup, both avian and mammalian Pmp22D and Pmp8G were able to adhere to all mammalian and avian cell lines to a similar degree, while only avian Pmp17G showed weak adhesion only to avian cells, suggesting a specific function in avian cells. OmcB, used as positive control, adhered strongly to all cell lines. Furthermore, pre-incubation of avian and mammalian cell lines with Pmp22D and Pmp8G significantly reduced the subsequent infection of all avian and mammalian *C. psittaci* strains analyzed, comparable to OmcB, indicating an essential role for these proteins in the infection process. The degree of infection rate was reduced by pre-incubation of the cells with these proteins; however, the infection was not completely prevented. This is likely due to the fact that chlamydiae harbor several Pmps and adhesins which may act on many different pathways, thus compensating the blockage of one infection route (Molleken et al., 2010; Becker and Hegemann, 2014). Considering all this, our data show that Pmp22D, Pmp8G, and OmcB are able to target some receptors, which are relevant for the infection of *C. psittaci* strains.

Interestingly, also avian Pmp17G was able to weakly to moderately reduce the infection of all strains, but only in avian cells, suggesting a specific interaction with a receptor or structure present on avian cells. The fact that the infection with all strains, avian and mammalian, was influenced by avian Pmp17G, indicates that Pmp17G may have blocked receptors targeted also by other Pmps on the bacterial surface. This suggests that some Pmps utilize the same cellular pathways, having redundant functions, while other Pmps might target specialized structures.

For the first time, this study investigates the different infectious potential of avian and mammalian *C. psittaci* strains in an *in vivo* model. Moreover, Pmp22D, Pmp8G, and OmcB were shown to be relevant adhesins, essential during the infection of *C. psittaci* strains in general, while Pmp17G could only target avian cells, suggesting a role in host adaptation. Our results support the hypothesis that the Pmp repertoires act in line with specific host factors to define the host tropism of *C. psittaci*.

## Data Availability Statement

The original contributions presented in the study are included in the article/Supplementary Material, further inquiries can be directed to the corresponding author/s.

## Author Contributions

AF conceived the study, performed and analyzed the experiments, and wrote the manuscript. CS conceived the study, performed and analyzed the egg infection experiments, and revised the manuscript. AT cloned expression vectors. MW performed the statistical analysis on expression data and produced heat maps. JH provided material for cloning and protein production and revised the manuscript. All authors contributed to the article and approved the submitted version.

## Conflict of Interest

The authors declare that the research was conducted in the absence of any commercial or financial relationships that could be construed as a potential conflict of interest.
