# Supplementary material for: Pmp Repertoires Influence the Different Infectious Potential of Avian and Mammalian Chlamydia psittaci Strains
Source: Front Microbiol. 2021 Mar 29;12:656209. doi: 10.3389/fmicb.2021.656209 (PMC8039305; doi:10.3389/fmicb.2021.656209)
Supplement: Supplementary file 1 [file Data_Sheet_1.pdf]

Supplementary Figures and Tables

Supplementary Figures

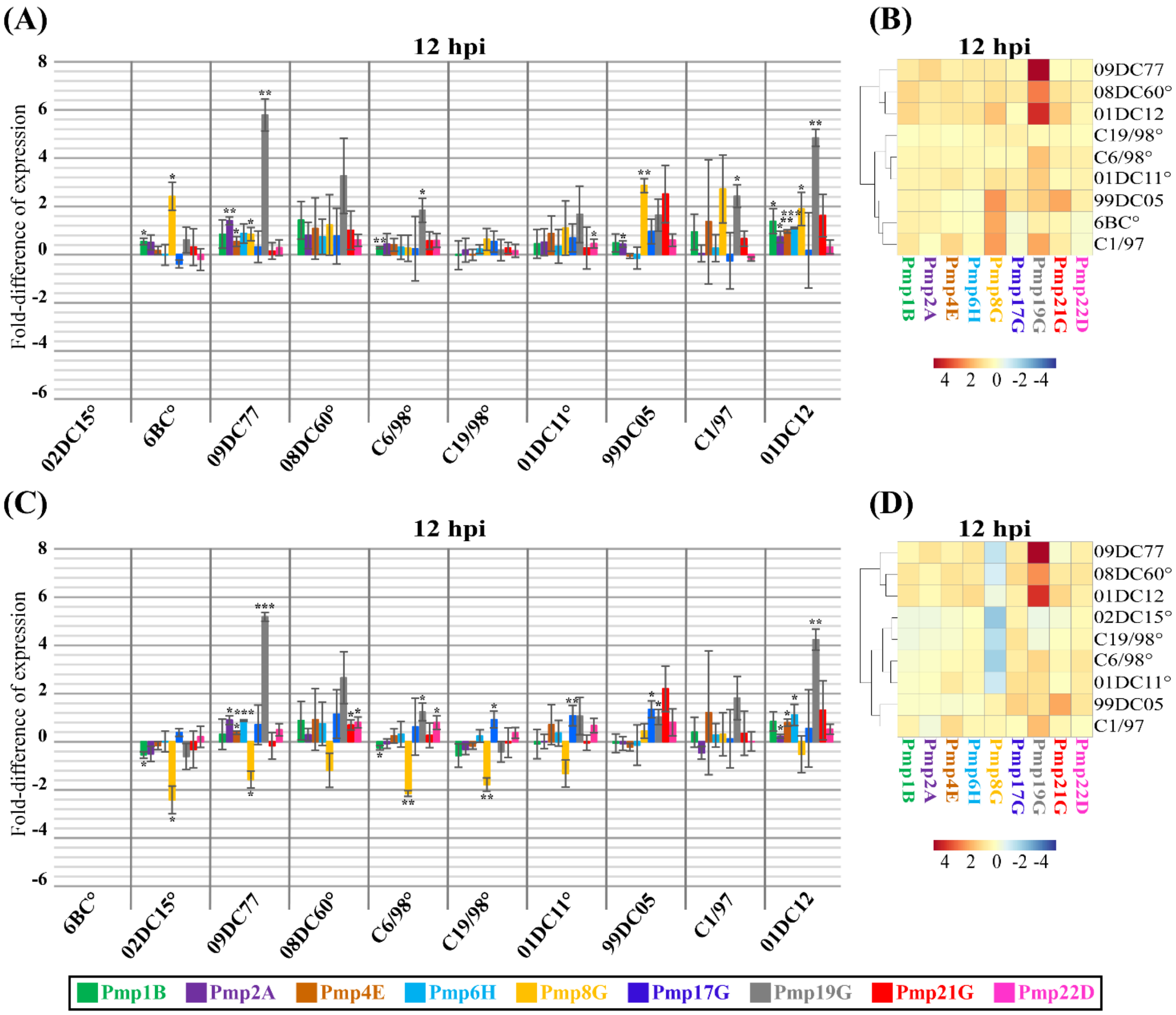

Supplementary Figure 1. Expression profiles of *pmp* genes in different *C. psittaci* strains infecting mammalian epithelial (BGM) cells at 12 hpi. Transcript levels of different color-coded *pmp* genes were measured by RT-qPCR at 12 hpi and expressed as relative fold-difference, compared to the respective *pmp* gene of mammalian 02DC15 **(A-B)** or avian 6BC **(C-D)** reference *C. psittaci* strains. °: *C. psittaci* strains belonging to genotype A. Relative expression levels represent the mean of three independent biological replicates (n=3). **(A-C)** Fold-difference of relative expression for each *pmp* in each strain is shown in color-coded columns, with the error bars representing the standard deviation of the mean. P-values were calculated using One-way Anova and post hoc t-test. *: p<0.05, **: p<0.001, ***: p<0.0001. **(B-D)** Expression heat maps illustrate the fold-difference of relative expression for each *pmp* in each strain. The strains are grouped according to their *pmp* expression pattern relative to those of 02DC15 or 6BC. Shades of red-colored cells represent folds of expression higher than the respective *pmp* gene in the reference strains, while shades of blue-colored cells represent folds of expression lower than the respective *pmp* gene in the reference strains.

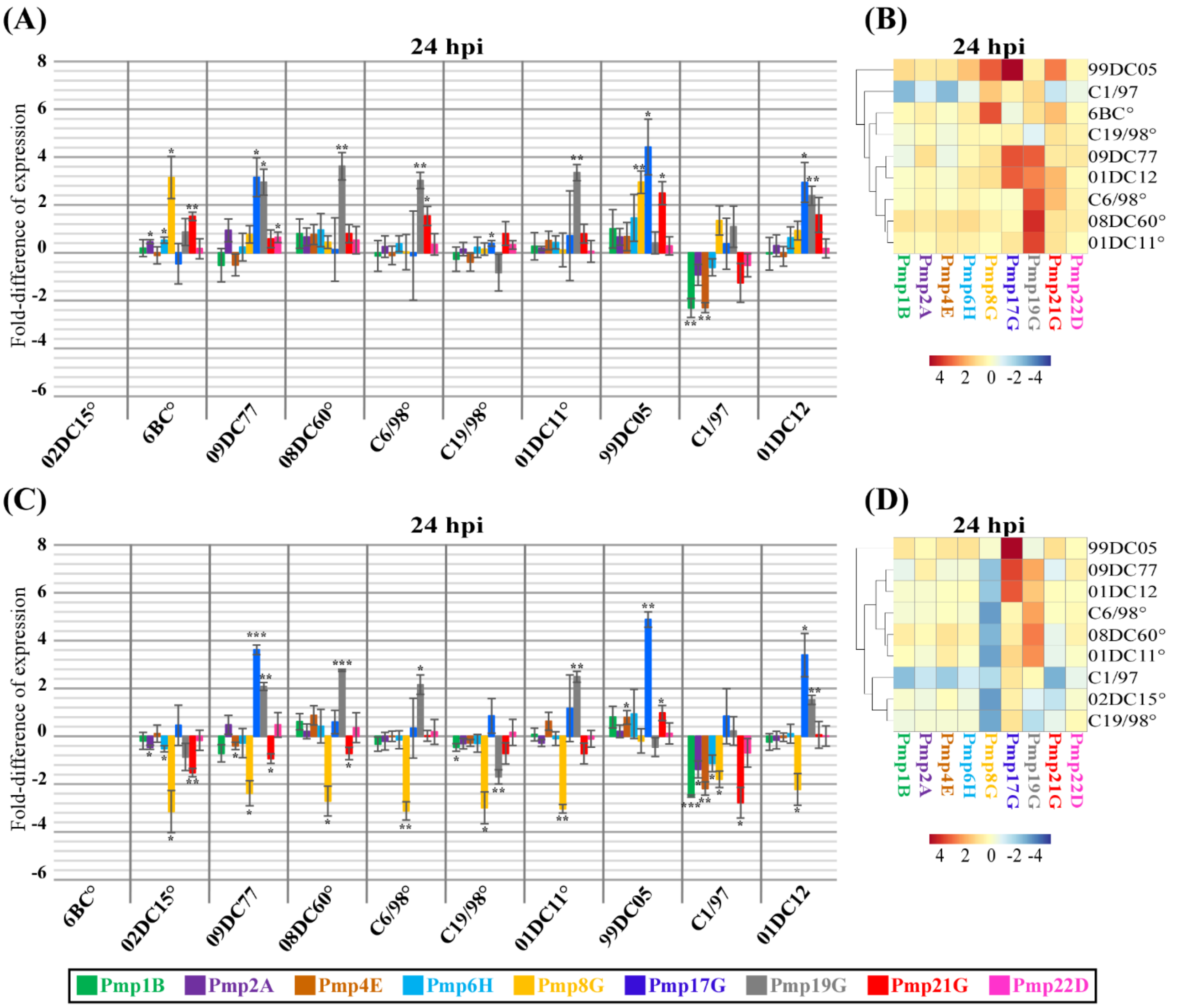

Supplementary Figure 2. Expression profiles of *pmp* genes in different *C. psittaci* strains infecting mammalian epithelial (BGM) cells at 24 hpi. Transcript levels of different color-coded *pmp* genes were measured by RT-qPCR at 24 hpi and expressed as relative fold-difference, compared to the respective *pmp* gene of mammalian 02DC15 **(A-B)** or avian 6BC **(C-D)** reference *C. psittaci* strains. °: *C. psittaci* strains belonging to genotype A. Relative expression levels represent the mean of three independent biological replicates (n=3). **(A-C)** Fold-difference of relative expression for each *pmp* in each strain is shown in color-coded columns, with the error bars representing the standard deviation of the mean. P-values were calculated using One-way Anova and post hoc t-test. *: p<0.05, **: p<0.001, ***: p<0.0001. **(B-D)** Expression heat maps illustrate the fold-difference of relative expression for each *pmp* in each strain. The strains are grouped according to their *pmp* expression pattern relative to those of 02DC15 or 6BC. Shades of red-colored cells represent folds of expression higher than the respective *pmp* gene in the reference strains, while shades of blue-colored cells represent folds of expression lower than the respective *pmp* gene in the reference strains.

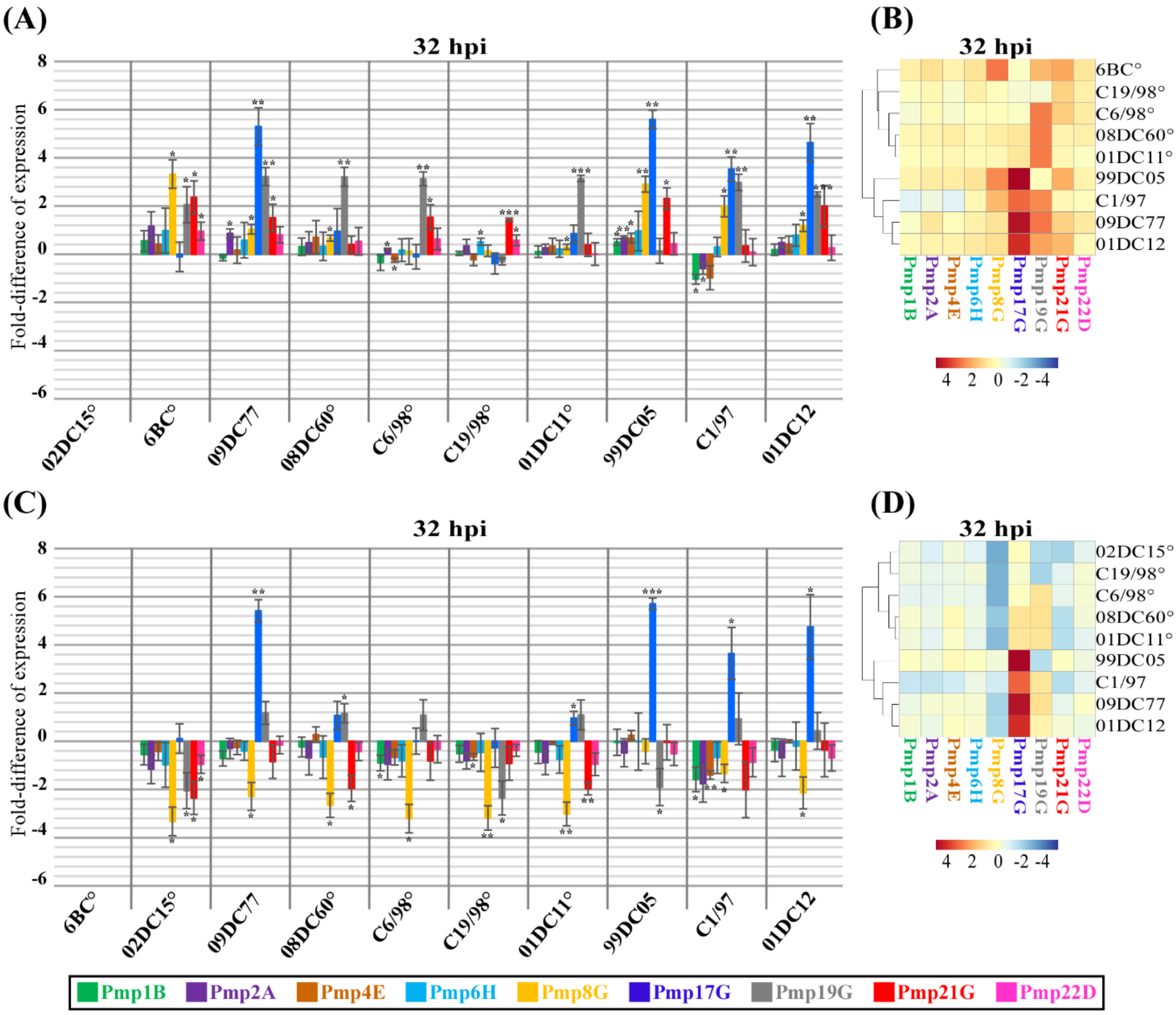

Supplementary Figure 3. Expression profiles of *pmp* genes in different *C. psittaci* strains infecting mammalian epithelial (BGM) cells at 32 hpi. Transcript levels of different color-coded *pmp* genes were measured by RT-qPCR at 32 hpi and expressed as relative fold-difference, compared to the respective *pmp* gene of mammalian 02DC15 **(A-B)** or avian 6BC **(C-D)** reference *C. psittaci* strains. °: *C. psittaci* strains belonging to genotype A. Relative expression levels represent the mean of three independent biological replicates (n=3). **(A-C)** Fold-difference of relative expression for each *pmp* in each strain is shown in color-coded columns, with the error bars representing the standard deviation of the mean. P-values were calculated using One-way Anova and post hoc t-test. *: $p<0.05$, **: $p<0.001$, ***: $p<0.0001$. **(B-D)** Expression heat maps illustrate the fold-difference of relative expression for each *pmp* in each strain. The strains are grouped according to their *pmp* expression pattern relative to those of 02DC15 or 6BC. Shades of red-colored cells represent folds of expression higher than the respective *pmp* gene in the reference strains, while shades of blue-colored cells represent folds of expression lower than the respective *pmp* gene in the reference strains.

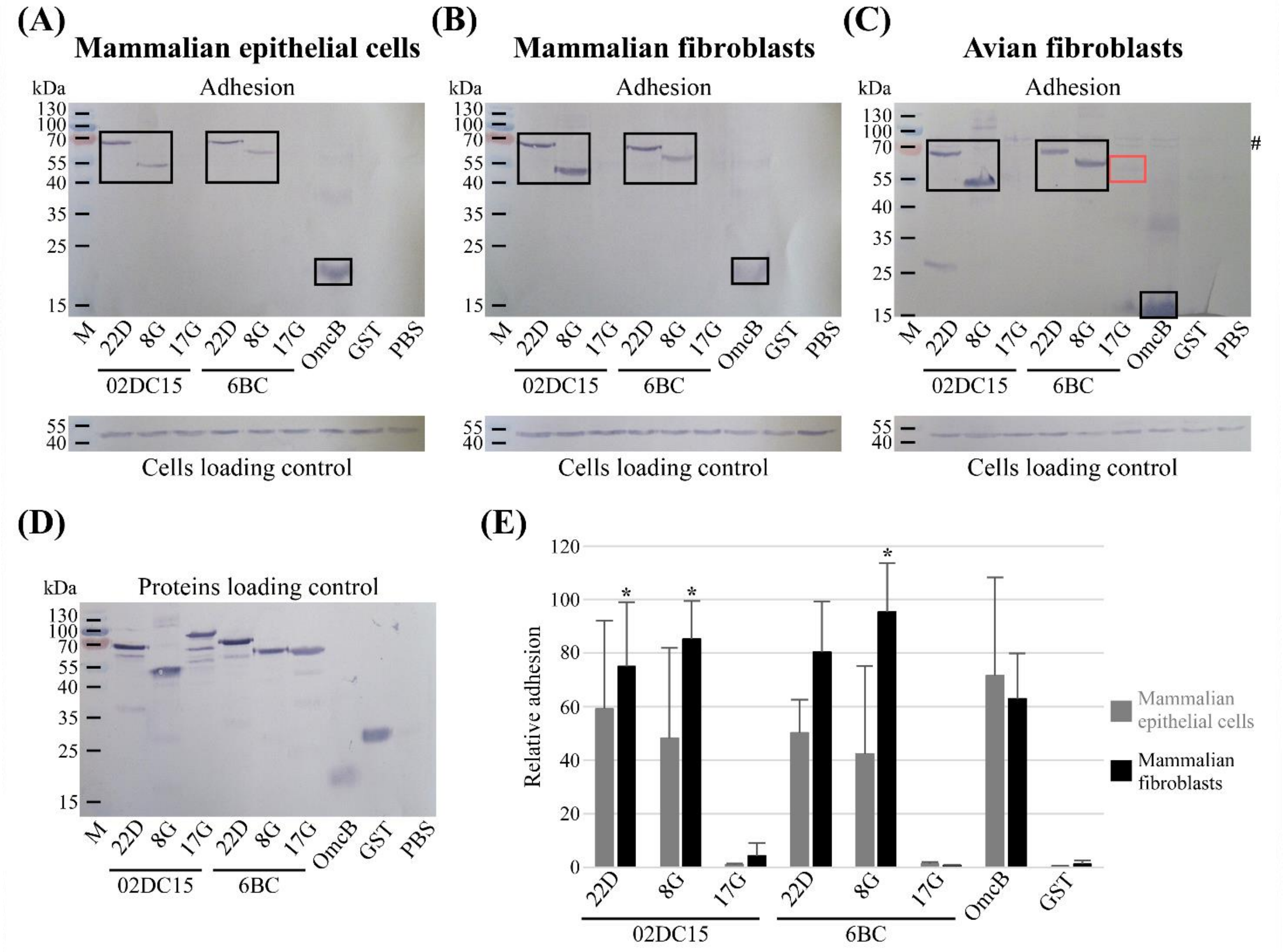

Supplementary Figure 4. Adhesion ability of different Pmps from mammalian and avian *C. psittaci* strains. (A-C) Representative western blots showing adhesion ability of 200 µg/ml soluble recombinant Pmp22D, Pmp8G and Pmp17G from mammalian *C. psittaci* 02DC15 and avian 6BC strains. Recombinant OmcB and GST were used as positive and negative control, respectively. Adhesion ability to confluent BGM mammalian epithelial cells **(A)**, McCoy mammalian fibroblasts **(B)** and UMNSAH/DF-1 avian fibroblasts **(C)**. **(D)** Input of soluble recombinant proteins was analyzed by immunoblotting prior to the assay. Anti-histidine antibody was used to determine the input of soluble recombinant proteins (protein loading control) and their binding ability (adhesion), anti-β actin antibody was used to verify the amount of cells in each sample (cells loading control). Black boxes mark the proteins, which are able to adhere to both mammalian and avian cells. Red box marks rPmp17G from avian strain 6BC, which is able to adhere only to avian cells. #: non-specific bands. **(E)** Comparison of adhesion intensity of different rPmps and control proteins to BGM mammalian epithelial cells (grey) and McCoy mammalian fibroblasts (black). The intensity of the bands was measured with ImageJ and expressed as a percentage of the input loading band of the respective protein. Relative adhesion represent the mean of three independent replicates (n=3). P-values were calculated using paired t-test, by comparing the relative adhesion of each Pmp to the two cell lines. *: $p<0.05$.

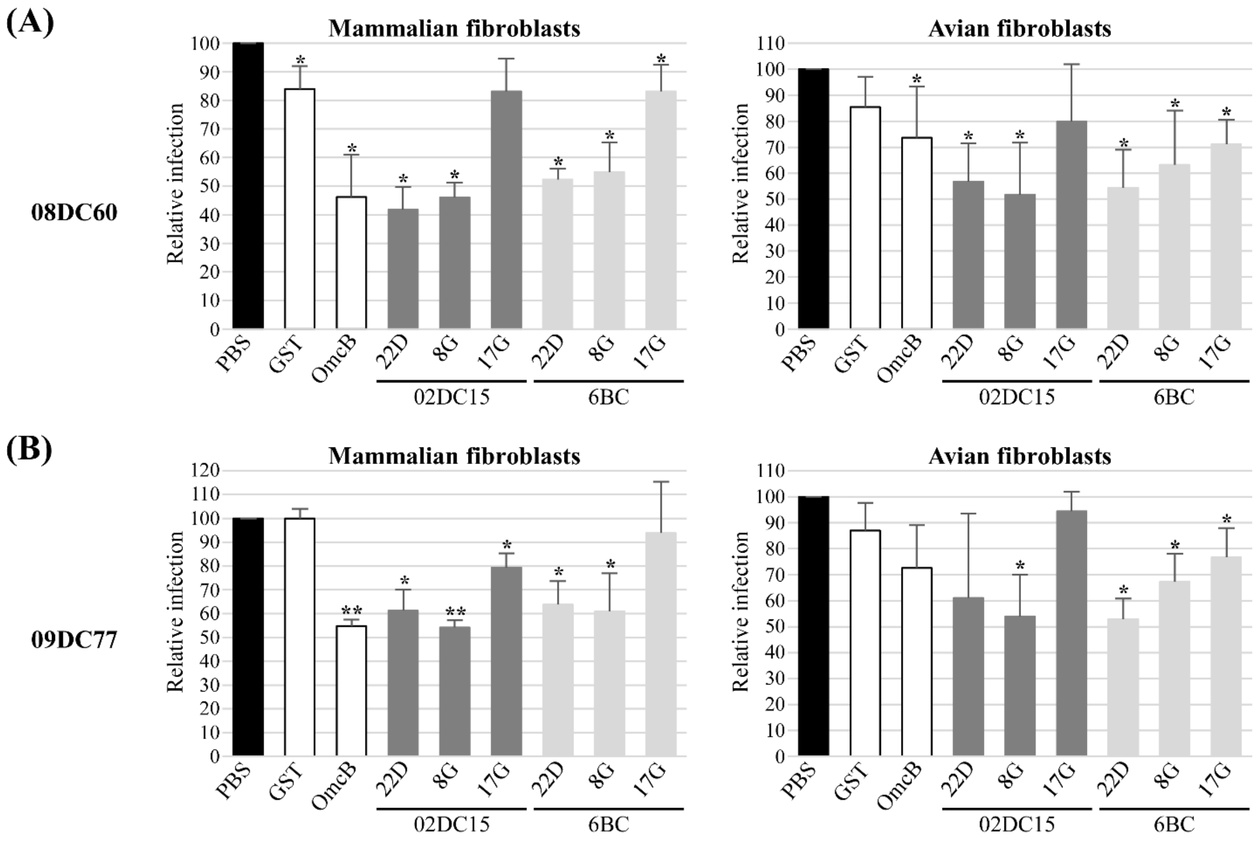

Supplementary Figure 5. Pmps from mammalian and avian *C. psittaci* strains show different relevance for *C. psittaci* infection. Mammalian (McCoy) and avian (UMNSAH/DF-1) fibroblasts were pre-incubated with PBS (black), controls recombinant GST and OmcB (white), or recombinant Pmps from mammalian 02DC15 (dark grey) and avian 6BC (light grey) *C. psittaci* strains for 1 hour prior to incubation with *C. psittaci* mammalian 08DC60 (**A**) or avian 09DC77 (**B**) strains. The numbers of inclusions were determined by observation of 10 microscopy fields for each condition. The infection rate for each sample is expressed as a percentage of the relative PBS-treated sample, set to 100%. The relative infection rates represent the mean of three independent experiments (n=3). P-values were calculated using paired t-test. *: $p<0.05$, **: $p<0.001$.

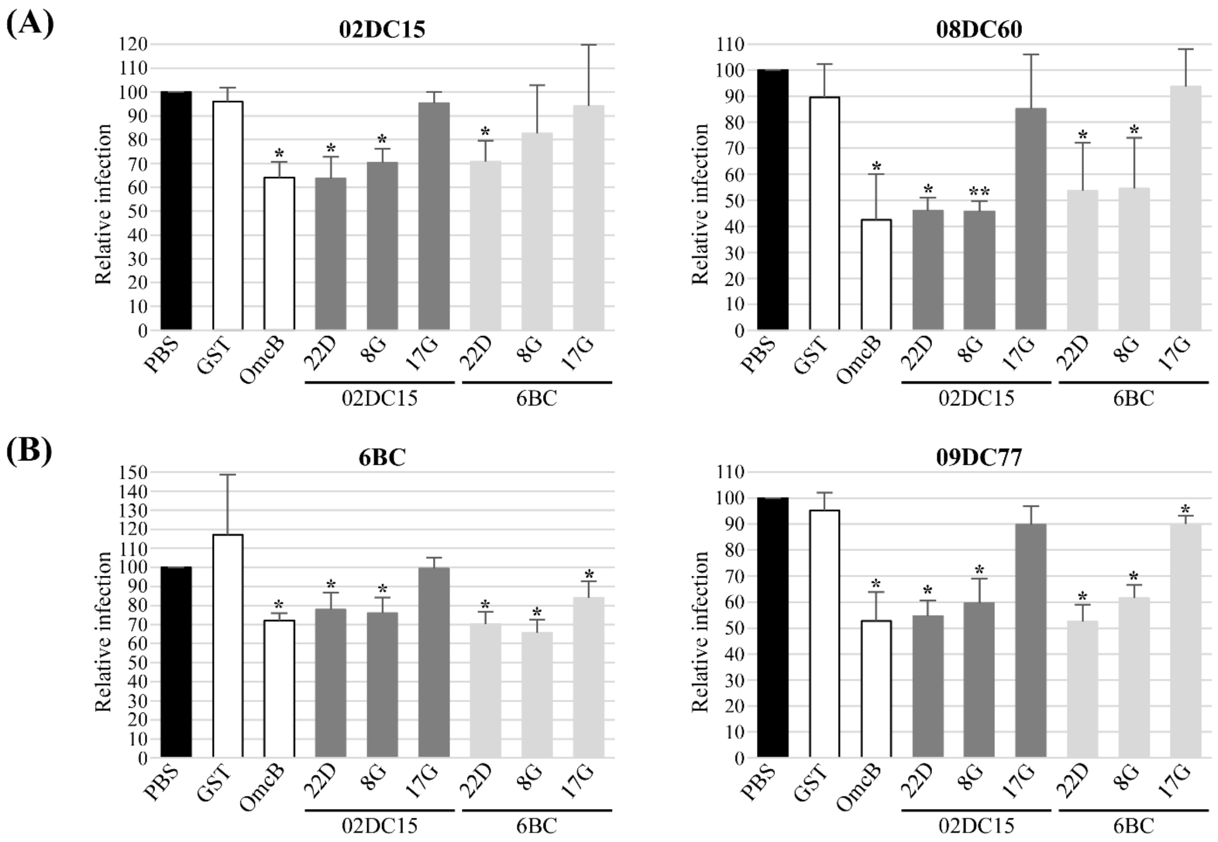

Supplementary Figure 6. Pmps from mammalian and avian *C. psittaci* strains show different relevance for *C. psittaci* infection in epithelial cells. Mammalian epithelial cells (BGM) were pre-incubated with PBS (black), controls recombinant GST and OmcB (white), or recombinant Pmps from mammalian 02DC15 (dark grey) and avian 6BC (light grey) *C. psittaci* strains for 1 hour prior to incubation with *C. psittaci* mammalian 02DC15 and 08DC60 (**A**) or avian 6BC and 09DC77 (**B**) strains. The numbers of inclusions were determined by observation of 10 microscopy fields for each condition. The infection rate for each sample is expressed as a percentage of the relative PBS-treated sample, set to 100%. The relative infection rates represent the mean of three independent experiments (n=3). P-values were calculated using paired t-test. *: p<0.05, **: p<0.001.

Supplementary Tables

Supplementary Table 1. Primers targeting *pmps* and control genes used for RT-qPCR expression analysis. *: primers from (Van Lent et al., 2016b).

Gene	Primer	Primer sequence (5'-3')	Amplicon size (bp)	Tm (°C)	GC (%)	Efficiency (%)	C. psittaci strains
**pmp1B**	pmpB_F1	CCGCCTTTCCCTAAAGGAGG	210	57.8	60.0	98.2	6BC, 09DC77, 02DC15, 08DC60, C6/98, C19/98, 01DC11, 99DC05, C1/97, 01DC12
	pmpB_R1	CGGGAAAACAGGTGGTGGAG		58.4	60.0		
**pmp2A**	pmpA-1 *	GTCGCCAGAGAAGGTGTTCC	280	58.0	60.0	105.4	6BC, 09DC77, 02DC15, 08DC60, C6/98, C19/98, 01DC11, 99DC05, C1/97, 01DC12
	pmpA_R1	TGCTCTTCCTACAGGGGTCTC		58.0	57.1		
**pmp4E**	pmpE2-1 *	GGGTTGAGTGGAGGGCATT	214	57.6	57.9	94.9	6BC, 09DC77, 02DC15, 08DC60, C6/98, C19/98, 01DC11, 99DC05, C1/97, 01DC12
	pmpE2_R1	CGCAGTGCAGGAGCCATATT		58.0	55.0		
**pmp6H**	pmpH_F3	CAGGAAACTTCCCGAAGAAAGCG	249	58.3	52.2	90.7	6BC, 09DC77, 02DC15, 08DC60, C6/98, C19/98, 01DC11, 99DC05, C1/97, 01DC12
	pmpH_R3	GCCAACACGAAGGTGTTCTTGT		58.2	50.0		
**pmp8G**	pmpG8_F2	AGCATAGATAGCCCCTCCACC	206	58.4	57.1	102.5	6BC, 09DC77, 02DC15, 08DC60, C6/98, C19/98, 01DC11, 99DC05, C1/97, 01DC12
	pmpG8_R2	GTGCCGTTGTCTCTAAAGGATCTC		57.2	50.0		
**pmp17G**	pmpG17_F1	ATTAGGGACGAGGGTTGCAC	315	57.1	55.0	108.0	6BC, 09DC77, 02DC15, 08DC60, C6/98, C19/98, 01DC11, 99DC05, C1/97, 01DC12
	pmpG17_R1	GGCGGAACGGGAAGTGTTAC		58.5	60.0		
**pmp19G**	pmpG19_F1-2	GCTGGGTATGCCTTAGGTGTC	214	57.7	57.7	86.2	6BC, 02DC15, C19/98
	pmpG19_R1	AACTAACGGAGCCTCAGCAC		57.2	55.0		
	pmpG19_F1-2	GCTGGGTATGCCTTAGGTGTC	214	57.7	57.7	101.2	99DC05

	pmpG19_R2	GGACTAATGGAGCTTCTACGCC		57.2	54.5		
	pmpG19_F5	GTGTGTATCTCCTATGCAGGG	185	54.7	52.4	101.5	09DC77, 08DC60, C6/98, 01DC11, C1/97
	pmpG19_R5	GAAAATCCTGAGACGCCTAAGG		55.4	50.0		
	pmpG19_F6	CGGAGAGGGCCCTACTACTTAC	251	58.2	59.1	108.1	01DC12
	pmpG19_R6	CAATGGCTTCTCCACCTGAGG		57.9	57.1		
**pmp21G**	pmpG21_F3	GCAAGCAGTGGTACCACAGAA	239	57.7	52.4	92.1	6BC, 09DC77, 02DC15, 08DC60, C6/98, C19/98, 01DC11, C1-97, 01DC12
	pmpG21_R3	TAATATCCCCGAGATCAGCGGTTA		57.1	45.8		
	pmpG21_F4	ACTCTTCTAAGGCAAAAGGTGGGG	252	59.0	50.0	110.5	099DC05
	pmpG21_R4	TCCCACCAGAGCCAAGATCG		59.2	60.0		
**pmp22D**	pmpD_F1	GTTAAAAGCACCGCCCCCTA	155	57.7	55.0	100.4	6BC, 09DC77, 02DC15, 08DC60, C6/98, C19/98, 01DC11, 99DC05, C1/97, 01DC12
	pmpD_R1	GTGAGTCTCAAGCAGGTGGT		57.0	55.0		
**tyrS**	tyrS_F1	GTGCCTAAAGTCGCACGTACG	242	58.4	57.1	98.3	6BC, 09DC77, 02DC15, 08DC60, C6/98, C19/98, 01DC11, 99DC05, C1/97, 01DC12
	tyrS_R1	ACTCCCTGGCCCATAGCAAT		58.8	55.0		
**gidA**	gidA_F1	GTAAGGGAGGGACAAACGGC	248	58.2	60.0	101.5	6BC, 09DC77, 02DC15, 08DC60, C6/98, C19/98, 01DC11, 99DC05, C1/97, 01DC12
	gidA_R1	CGGGACGTTTATGCGTGGTT		58.2	55.0		

Supplementary Table 2. Cloning primers for *pmps* and control genes and resulting protein fragments.

Gene	*C. psittaci* strain	Primer sequence (5'-3')	Protein fragment (aa)	Molecular weight (kDa)	
				Calculated	Apparent
**pmp22D**	02DC15	AAATAATTTTGTTTAACTTTAAGAAGGAGATATACATATG**ATTGCTCACAACGGGAAAGT**	690-1240	58.7	70
		TTTGTTAGCAGCCGGATCTCAGTGGTGGTGGTGGTGGTG**CTGTTGTTGTTTTAAAGATCG**			
**pmp22D**	6BC	AAATAATTTTGTTTAACTTTAAGAAGGAGATATACATATG**GCTTTGTTTGGGTTTGATGC**	651-1214	59.6	75
		TTTGTTAGCAGCCGGATCTCAGTGGTGGTGGTGGTGGTG**TAACTTATAACTCGTAGGCTT**			
**pmp8G**	02DC15	AAATAATTTTGTTTAACTTTAAGAAGGAGATATACATATG**TCTCTCATGTTTCGCTCCAA**	51-450	43.5	50
		TTTGTTAGCAGCCGGATCTCAGTGGTGGTGGTGGTGGTG**GCTATTAGGAACTAGGGAATT**			
**pmp8G**	6BC	AATAATTTTGTTTAACTTTAAGAAGGAGATATACATATG**ACGTATATCTTATCTGATGAC**	40-530	53.5	65
		CTTTGTTAGCAGCCGGATCTCAGTGGTGGTGGTGGTGGTG**CCATAGGCTATTAGGAACTA**			
**pmp17G**	02DC15	AATAATTTTGTTTAACTTTAAGAAGGAGATATACATATG**AAAAACGTTGTCTTCTCTGGC**	40-600	58.0	85
		TTTGTTAGCAGCCGGATCTCAGTGGTGGTGGTGGTGGTG**GACTTTTCCACTCGAGTCTTT**			
**pmp17G**	6BC	AATAATTTTGTTTAACTTTAAGAAGGAGATATACATATG**TCAATTATCTTCCAAGGCAAT**	30-470	46.2	60
		TTTGTTAGCAGCCGGATCTCAGTGGTGGTGGTGGTGGTG**AATAGCAGAACACCATAAGCT**			
**OmcB**	02DC15	AATAATTTTGTTTAACTTTAAGAAGGAGATATACATATG**AGTACTGAAAACTCAGATGAC**	40-150	12.3	20
		TTTGTTAGCAGCCGGATCTCAGTGGTGGTGGTGGTGGTG**AAGCTGTTGAGTAATCACAAC**			
**GST**	-	AATAATTTTGTTTAACTTTAAGAAGGAGATATACATATG**TCCCCTATACTAGGTTATTGG**	1-218	25.4	30
		CTTTGTTAGCAGCCGGATCTCAGTGGTGGTGGTGGTGGTG**TTTTGGAGGATGGTCGCCAC**			

Supplementary Table 3. Identity of *pmps* from different *C. psittaci* strains to the respective *pmp* gene of 02DC15, used as reference strain. Identities are calculated on the sequence coverage of 02DC15 *pmps*, using Blast and Geneious alignments. Color-coded boxes indicates the percentage of sequence coverage in the alignments: white boxes (100%), green boxes (90-99%) and orange boxes (80-90%). X: missing or incomplete gene sequences.

***C. psittaci* strains**	**Pmp genes (% of identity to 02DC15 *pmps*)**																				
**02DC15**	**1B**	**2A**	**3E**	**4E**	**5E**	**6H**	**7G**	**8G**	**9G**	**10G**	**11G**	**12G**	**13G**	**14G**	**15G**	**16G**	**17G**	**19G**	**20G**	**21G**	**22D**
**6BC**	100	100	100	99	100	100	100	99	100	100	100	100	99	100	99	100	99	99	99	99	100
**09DC77**	98	99	91	98	99	95	94	99	96	99	99	92	95	X	89	98	93	X	92	92	99
**08DC60**	100	100	100	99	100	100	100	99	100	100	99	99	99	99	99	99	100	98	99	99	100
**C6/98**	99	100	99	99	100	100	100	99	100	99	100	99	99	X	98	100	100	99	99	98	100
**C19/98**	99	100	99	99	100	100	100	99	100	99	100	99	99	X	95	100	100	99	100	99	100
**01DC11**	100	100	100	99	100	100	100	99	100	100	100	99	99	99	99	100	100	98	99	99	100
**99DC05**	94	99	97	96	99	97	94	99	93	99	99	93	95	94	94	99	97	83	88	88	99
**C1/97**	97	99	90	95	99	95	95	99	99	99	99	X	X	X	97	99	93	99	97	99	92
**01DC12**	98	99	90	98	99	95	93	98	96	99	99	92	90	X	X	99	93	82	81	88	99
